# Facile synthesis of water-soluble carbon nano-onions under alkaline conditions

**DOI:** 10.3762/bjnano.7.67

**Published:** 2016-05-27

**Authors:** Gaber Hashem Gaber Ahmed, Rosana Badía Laíño, Josefa Angela García Calzón, Marta Elena Díaz García

**Affiliations:** 1 Department of Physical and Analytical Chemistry, Faculty of Chemistry, University of Oviedo, c/Julián Clavería, 8. Oviedo, 33006, Spain; 2 Chemistry Department, Faculty of Science, Damanhour University, Damanhour, Egypt

**Keywords:** carbon dots, carbon onions, metal-ion sensing, photoluminescence, thermal carbonization

## Abstract

Carbonization of tomatoes at 240 °C using 30% (w/v) NaOH as catalyst produced carbon onions (C-onions), while solely carbon dots (C-dots) were obtained at the same temperature in the absence of the catalyst. Other natural materials, such as carrots and tree leaves (*acer saccharum*), under the same temperature and alkaline conditions did not produce carbon onions. XRD, FTIR, HRTEM, UV–vis spectroscopy, and photoluminescence analyses were performed to characterize the as-synthesized carbon nanomaterials. Preliminary tests demonstrate a capability of the versatile materials for chemical sensing of metal ions. The high content of lycopene in tomatoes may explain the formation of C-onions in alkaline media and a possible formation mechanism for such structures was outlined.

## Introduction

In the last twenty years, carbon based nanomaterials have received much research attention not only from a basic perspective but also from a practical point of view due to their use in a range of applications such as energy storage, tribology, electronics, medicine, catalysis and sensors. The more popular and extensively investigated carbon-based nanomaterials include carbon dots (C-dots), fullerenes, nanotubes and graphene, while others, such as nanodiamonds and carbon onions, stayed forgotten for a long time, in spite of the fact that these carbon nanoparticles (C-NPs) were discovered before the former [1].

C-dots are a special class of carbon nanoparticles that have interesting practical advantages such as low toxicity, relatively small size (≤10 nm), chemical stability, high solubility in water and easy synthesis. Besides, owing to their remarkable photoluminescence (PL) properties, such as broad excitation spectra, tunable emission wavelength and stable PL, high stability against photobleaching, C-dots are attracting considerable attention in analytical sensing, bioimaging, photo-reduction of metals and biomedical applications [2–6].

C-dots may be straightforwardly synthetized via two approaches: a) from fine carbon structures (such as multi-wall nanotubes and graphene) by top-down methods and b) by bottom-up approaches from chemical precursors (such as glucose, citrate, ethylenediaminetetraacetic acid) or from natural products (usually vegetables). Recently, using the bottom-up approach, we prepared C-dots based on the thermal carbonization of a mixture of nitrogen-containing organic compounds, ethyleneglycol bis(2-aminoethyl ether)-*N*,*N*,*N*′,*N*′-tetraacetic acid (EGTA) and Tris, thus providing them not only with surface hydroxy but also with amino groups. These C-dots were successfully employed for sensitive detecting 4-nitrophenol in water [7]. In another work, with the aim to provide surface boronic groups, we prepared C-dots by hydrothermal treatment of a mixture of 6-bromohexylboronic acid, polyethyleneglycol bis(3-aminopropyl)-terminated (PEGA) and 1,2-aminopropane (DPA) at 180 °C. We could observe that during the thermal reaction, the boronic groups were unstable and tended to leave the surface of the C-dots. Using these C-dots, reliable determination of tannic acid in wines was achieved with a detection limit of 0.018 mg·L^−1^ [8].

Carbon nano-onions (C-onions) are another kind of carbon nanoparticles that exhibit outstanding chemical and physical properties. C-onions are spherical carbon shells enclosed within one another (multi-layered fullerenes) with diameters ranging from 3 to 50 nm [9], depending on the method of synthesis. C-onions have found applications as materials for tribology due to their low friction [10]. Polymers doped with C-onions exhibit increased thermal resistance and can be used as microwave absorbing filters due to the C-onions ability to absorb electromagnetic radiation in the 26–37 GHz range [11]. Also, C-onions have attracted attention for batteries and supercapacitors, as active materials and/or dispersible conductive additives [12–13]. Usually C-onions are obtained by using sophisticated technologies, such as vacuum annealing of nano-diamond precursors [14–15], nano-diamond annealing in inert gases [16], arc discharge in presence of metal nanoparticles [17], high-energy laser excitation of ethylene at high temperatures [18] and chemical vapor deposition using catalysts [19]. There are some excellent reviews devoted to C-onions and their chemistry and applications [20–21]. Due to the intricate processes, the running costs and high investment for their synthesis, the use of C-onions for analytical applications is still unexplored. Here, we describe the synthesis and characterization of C-nanoparticles obtained by thermal carbonization at 240 °C of tomatoes, carrots and tree leaves samples as C-source in absence and in presence of 30% (w/v) NaOH. We discovered that the use of NaOH as catalyst favored the formation of C-onions when tomatoes were used as C-source and that intermediate carbon nanostructures were formed when carrots or tree leaves were used. On the basis of the morphologies and spectral characteristics of these structures the formation mechanism of C-onions is proposed. Finally, a preliminary test on the use of such C-onions as sensing materials for metal ions is outlined.

## Results and Discussion

**Figure 1:**
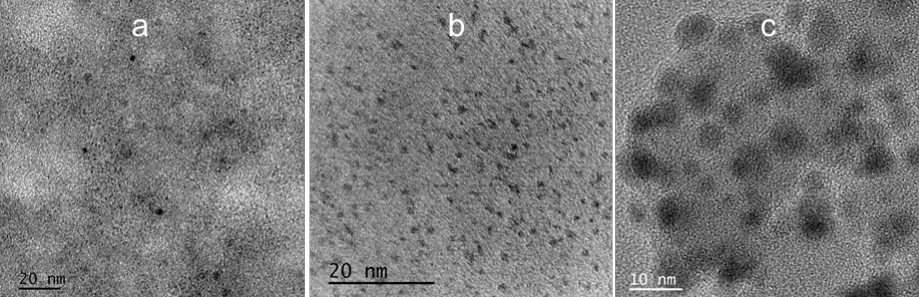
HRTEM images of C-dots obtained by carbonization in aqueous environment, using as carbon source a) tomatoes, b) carrots and c) tree leaves.

In Figure 1a the HRTEM image reveals that the tomato C-dots synthetized by conventional carbonization are mostly spherical, with diameters well below 1 nm as also can be observed for C-dots from tree leaves (Figure 1c). In Figure 1b, the HRTEM image shows that C-dots from carrots are also spherical but with diameters around 5 nm.

**Figure 2:**
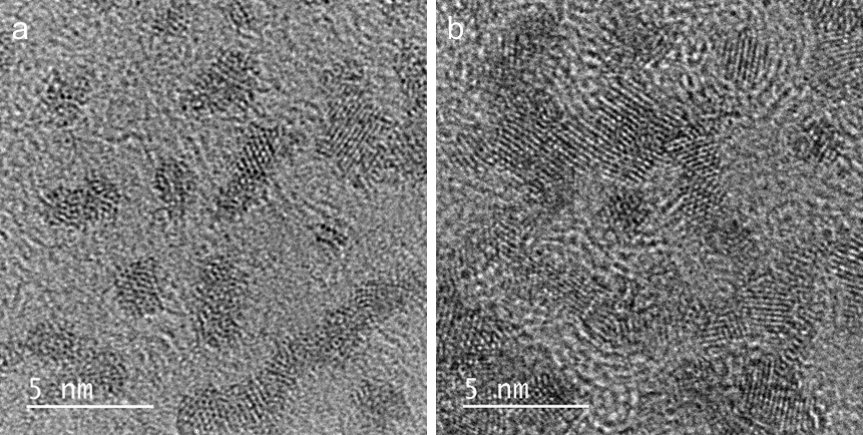
HRTEM images of C-dots obtained by carbonization in NaOH 30% (w/v), using as carbon source a) carrots, b) tree leaves.

When using a NaOH 30% (w/v) media the C-NPs exhibited a different morphology, as can be observed in Figure 2a,b for carrots and tree leaves, respectively. C-dots with no well-defined structure were obtained, in which a crystal order could be observed.

**Figure 3:**
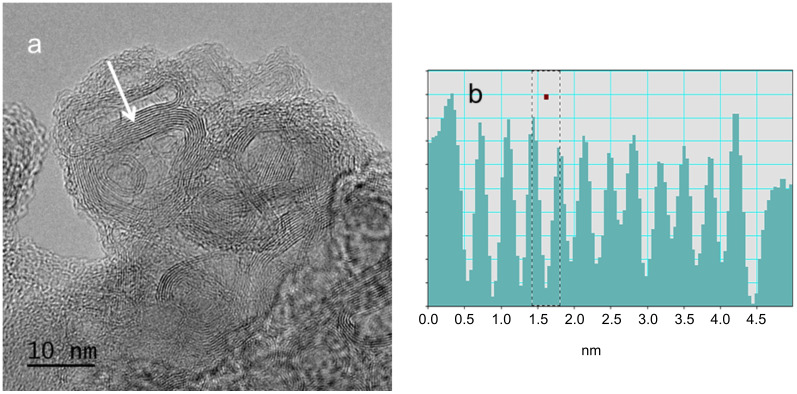
a) HRTEM image of C-onions obtained by carbonization in NaOH 30% (w/v), using tomatoes as carbon source; b) intensity profile (arbitrary units) measured over the white arrow marked in a).

In the case of tomatoes as carbon source, C-NPs clearly show an onion-like structure (Figure 3a) for which the interlayer spacings were determined to be about 0.3 nm by EDX (area highlighted in Figure 3b). This spacing is slightly smaller than the interlayer spacing of (002) planes of graphite [22]. It is worth to mention that along with the C-onions some buckled structures can be observed. Assuming that C-onions grow from the inside to the outside, the buckled sheets can be attributed to carbon layers that do not fit during the growing process of the C-onions. In other words, the layers may not remain spherical during the growth. Continuum mechanical shell models have been applied to investigate the growth limit and buckling patterns of C-onions [23]. To the best of our knowledge, the results shown here, demonstrate for the first time, the possibility of obtaining C-onions from green C-sources just by modifying the carbonization conditions using sodium hydroxide as catalyst.

**Figure 4:**
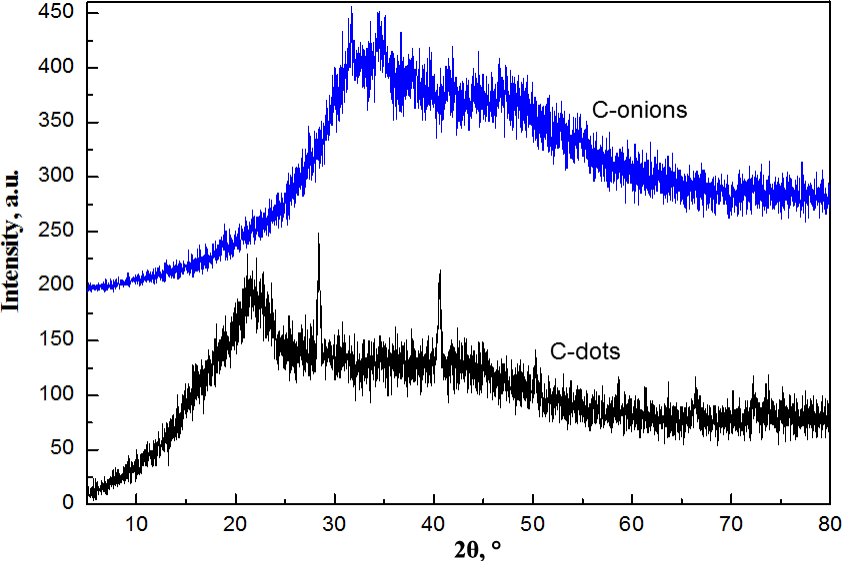
XRD patterns of C-NPs obtained from tomatoes through carbonization in aqueous (C-dots) and NaOH 30% (w/v) (C-onions) media.

Figure 4 represents the XRD pattern of the C-NPs produced by the one-step thermal carbonization of tomatoes. For C-dots, a featureless reflection band centered at 2θ = 21.68° corresponds to the diffraction of graphite [002] crystal planes and a weak broad peak at about 2θ = 43° corresponds to the {100/101} set of crystal planes [24] of graphite.

The interlayer spacing was determined by using Bragg’s equation and was found to be 4.32 Å. The mean crystallite size, *L*_c_*,* was determined for the [002] band using Scherrer's equation (Equation 1):

[1]
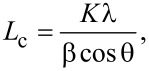


where λ is the X-ray wavelength (1.5405 Å), β is the broadening of the diffraction peak measured at half its maximum intensity (in radians), θ is the scattering angle and *K* is the Scherrer’s constant, which varies with the shape of the crystallites, from 0.89 for spherical to 0.94 for cubic particles [25]. Considering that this constant is set to 0.9 for particles of unknown size, the *L*_c_ was estimated to be 7.4 Å for the C-dots. There is a comparatively large difference to the values reported for graphite (3.34 Å), the reason behind which may be poor crystallization and/or formation of misoriented (turbostratic) carbon structures [26]. The XRD spectra of C-dots from carrots and tree leaves showed a similar pattern (Figure S1, Supporting Information File 1).

In the case of C-onions, the diffraction pattern clearly showed that the [2] graphite peak disappeared while new peaks appeared. We ascribed the peak at 33° to the cubic phase of K_2_O [JCPDS card no. 23-493], taking into account the high concentrations of potassium in these vegetable samples [27]. The peak at 46° can be ascribed to a rhombohedral phase of graphite corresponding to a [101] reflection, probably due to the introduction of stacking faults in the crystallites with hexagonal stacking upon the NaOH treatment. The XRD for the C-NPs obtained for tomatoes, carrots and tree leaves by carbonization in NaOH 30% (w/v) media are shown for comparison (Figure S2, Supporting Information File 1). As can be seen, a similar pattern XRD pattern was observed for the three systems, which indicated that similar crystal phases were obtained.

**Figure 5:**
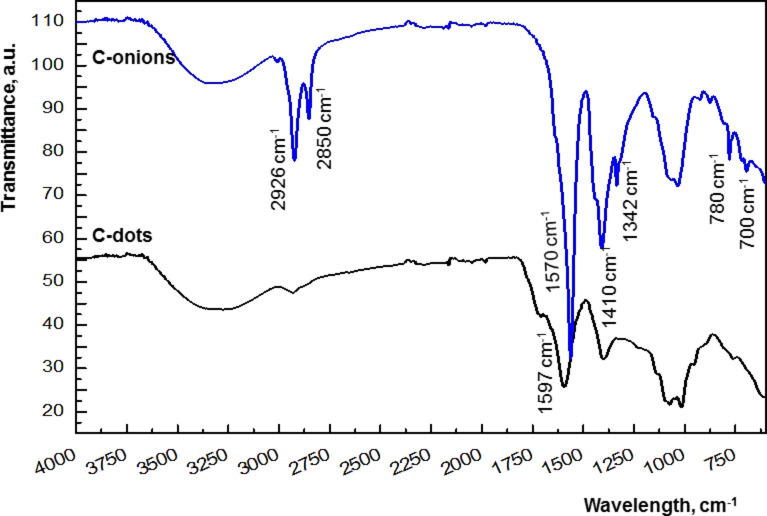
FTIR spectra of C-dots and C-onions obtained by carbonization of tomatoes in aqueous solution and NaOH 30% (w/v), respectively.

The functional groups of the C-dots were determined by FTIR analysis. In Figure 5, it can be observed that C-onions exhibited very sharp bands at 2926 and 2850 cm^−1^ due to C–H stretch (methylene/methyl) vibrations and aldehyde C–H stretching, respectively, while these bands were very weak for C-dots.

The presence of a weak shoulder band at 1720 cm^−1^ in the C-dots spectrum suggested the presence of saturated aldehyde C=O stretching, while this band was not observed in C-onions. On the other hand, the bands at 1170–1600 cm^−1^ assigned to C=C stretching and the peak at 1410 cm^−1^ attributed to O–H bending vibrations (carboxylic acid) can be observed in both types of nanoparticles. The medium-intensity band at 1342 cm^−1^ observed in C-onions, but not in C-dots, was ascribed to δ_S_ C–O–H absorption [28–29]. The peak at 1570 cm^−1^ observed for C-onions can be ascribed to the phenol C=C phenol ring stretching. The intense bands observed at 2926 and 2851 cm^−1^ are characteristics of saturated asymmetric stretching vibrations CH_str_ (sp^3^) and symmetric stretching vibrations of –R–CH_2_=O (aldehyde group), respectively. These two peaks can be hardly observed in the C-dots spectra. The peaks at 1010–1070 cm^−1^ are from characteristic C–O stretchings of primary alcohols. The peaks at 700 and 780 cm^−1^ observed in C-onions, but not in C-dots, are ascribed to –OH out-of-plane bending vibrations of alcohols. Again, these results demonstrated the possibility of obtaining water-soluble C-onions (due to the presence of carboxy and hydroxy groups) by carbonization in basic media.

The FTIR spectra of the C-NPs obtained when carrots and tree leaves were carbonized in a NaOH 30% (w/v) media are shown in comparison (Figure S3, Supporting Information File 1). As can be observed, the functional groups of all nanoparticles seemed to be the same, being the only difference the higher intensity of the 2926 and 2850 cm^−1^ bands for C-onions from tomatoes compared with those of carrots and tree leaves. This may suggest that also C-onions were formed when carrots and tree leaves were carbonized in a NaOH 30% (w/v) media. Taking into account that with respect to the starting mass of tomatoes, the C-onions average yield was only (2 ± 0.5)% and that no C-onions could be observed in the HR-TEM images of tree leaves and carrots, it was reasonable to assume that the C-onions yield from carrots and tree leaves was less than 2%.

The PL spectra of C-dots obtained in aqueous media from tomatoes, at different excitation wavelengths, are shown in Figure S4 of Supporting Information File 1. The maximum emission wavelength depends on the excitation wavelength and is shifted from 438 to 456 nm (Δλ_em_ = 18 nm) by changing the excitation wavelength from 300 to 370 nm (Δλ_ex_ = 70 nm). It was found that also C-onions obtained in basic media exhibited excitation-dependent PL (Figure S4, Supporting Information File 1): The emission maxima shifted from 427 to 440 nm (Δλ_em_ = 13 nm) as the excitation wavelength moved from 300 to 350 nm (Δλ_ex_ = 50 nm). PL quantum yield of C-dots excited at 362 nm resulted to be 1.32% while that of C-onions excited at 328 nm was 1.63%. These values were similar or even higher than those obtained for carbon dots prepared by the same route of synthesis using different starting materials (Table S1, Supporting Information File 1).

**Figure 6:**
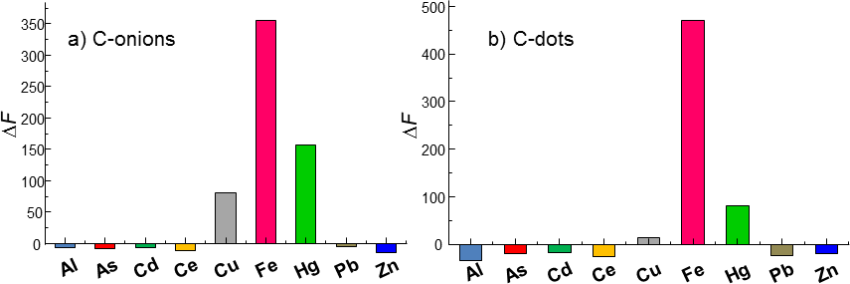
Effect of some metals on the photoluminescence spectra of C-NPs at pH 6. a) C-onions measured at λ_ex_ = 328 nm. Slit width of emission and excitation was 10 and 20, respectively. b) C-dots measured at λ_ex_ = 362 nm. The slit widths of emission and excitation were both 20 nm. The concentration of metals was 10 ppm in all cases.

To the best of our knowledge, this is the first report on the synthesis of water-soluble fluorescent C-onions. In order to check the analytical potential of these C-onions as sensing material, their PL response was determined upon exposure to different metal ions and the results were compared with those obtained using C-dots. In Figure 6a, the quenching effect, expressed as Δ*F* = *F*_0_ − *F*, where *F*_0_ and *F* are the PL of the C-onions in absence and presence of a given metal ion, respectively, is shown. As can be seen, Cu(II), Fe(III) and Hg(II) quenched the PL of the C-onions, Fe(III) being the strongest quencher. In the case of C-dots (Figure 6b), Fe(III) was the main quencher and Hg(II) also quenched to a lesser extent. Consequently, the selectivity against metal ions of the C-onions seemed to be lower than that of C-dots. These results may be explained on the basis of the C-onions structure, with many concentric surfaces exposed for binding, which enhanced the probability for quenching.

**Figure 7:**
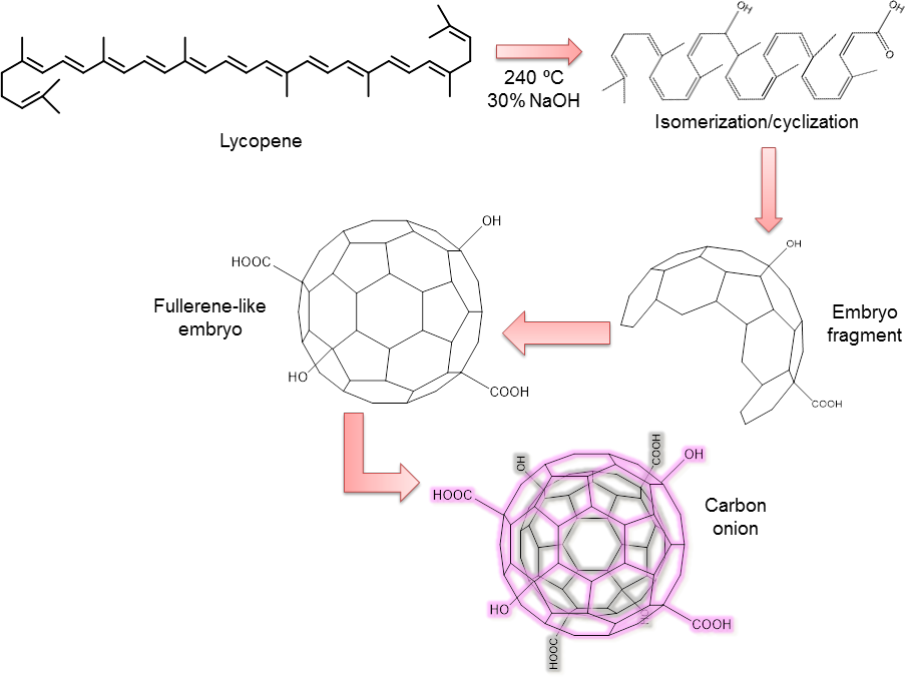
Hypothetical growth of carbon onions from lycopene.

Results obtained from the characterization of C-onions suggested that a possible mechanism for the formation of C-onions may be related to the presence of polyene molecules in tomatoes, carrots and leaves, particularly in the case of tomatoes with a high content of lycopene. Under elevated temperatures, oxygen and/or extremes in pH, lycopene molecules may undergo isomerization and oxidation [30–31] and/or break down into small fractions [32]. These products may form fullerene-like embryos which then reorganized into spherical particles composed of concentric graphitic layers (Figure 7), in order to minimize the surface energy of the newly formed edge planes of graphite [9]. Although similar open/closed geodesic structures have been used as models for quantum chemical modelling the growth and the molecular and electronic structures of fullerenes and carbon onions [33–34], the atomic arrangements inducing curvature are still not fully understood. In the case of carrots and tree leaves, the reorganization into onions was not so clear as in the case of tomatoes, although it may be related to the low content of lycopene in these vegetables. We suggest that further investigation of the factors affecting such reorganization must be performed, such as the time of heating, the amount of NaOH or the temperature used, which also will help to improve the yield of the synthesis.

## Conclusion

C-onions obtained by conventional synthetic methods such as arc-discharge and chemical vapor deposition are insoluble in water, which restricts their use in analytical, biological and biomedical applications. Our results showed that water-soluble C-onions could be prepared by a simple carbonization method using tomatoes as carbon source and 30% (w/v) NaOH as catalyst. We are aware of the danger of drawing conclusions from preliminary data. However, we sense that this synthetic procedure has the necessary characteristics for further studies and development of tailor-made water-soluble C-onions from polyene molecules with different functionalities in basic media. Given their closed structure, water soluble C-onions could be loaded with specific molecules or drugs in bio-medical applications while the delivery process could be monitored thanks to their exceptional PL properties. The field is open.

## Experimental

### Chemicals and reagents

All the reagents used were highly pure analytical grade chemicals and used without further purification. NaOH 30% (Prolabo, http://www.vwr.com) was used in the synthesis step. In subsequent steps the following reagents were used: quinine sulfate, H_2_SO_4_, and Cu(NO_3_)_2_·3H_2_O were purchased from Prolabo, FeCl_3_, ZnCl_2_, Na_2_HPO_4_ and citric acid were purchased from Sigma-Aldrich, AlCl_3_·6H_2_O and Pb(Ac)_2_·3H_2_O were purchased from Montplet & Esteban S.A., Barcelona (http://www.montplet.es/), Ce(NO_3_)_3_·6H_2_O was purchased from Fluka, Switzerland, HgCl_2_ was purchased from Porus (http://poruslabs.com/), and CdCl_2_ was purchased from The British Drug Houses Ltd., England. Stock solutions containing 100 ppm of the metal were prepared by dissolving the appropriate amount of each salt in distilled/deionized water. Tomatoes (type “Rama”) and carrots were purchased from a local market. Tree leaves (*acer saccharum*) were taken from the faculty garden.

### Synthesis of carbon nanoparticles

C-nanoparticles were synthesized by a thermal carbonization method using tomatoes, carrots and tree leaves as green carbon sources. Typically, after thoroughly cleaning, the starting material was grinded in small pieces and about 30–50 g was put into dried and cleaned crucibles. In order to prepare C-dots, the homogenized sample was carbonized in a muffle furnace directly at 240 °C in atmospheric oxygen for 2 h. The residue was then dissolved in about 25 mL Milli-Q water, filtered through 0.45 μm nylon filter and the solution was purified by dialysis through a dialyzer tube (MWCO, 3.5 KDa) for 3 days.

For the synthesis of C-onions 5 mL NaOH 30% (w/v) was added to the crucible containing the homogenized sample and then transferred into a muffle furnace and heated to 240 °C in atmospheric oxygen for 2 h. A yellow-brown solid was obtained when carbonizing the samples in the presence of NaOH 30%. The color is probably due to the partial oxidation of graphene to graphene oxide during the process [35]. The water-soluble part of the residue was extracted by dissolution in 100 mL deionized water, then filtrated through normal filter papers and followed by nylon filters (0.45 μm). The filtered solution was then purified through dialyzer tube (MWCO, 3.5 kDa) for 3 days. Each purified solution was divided into two aliquots, the first one was dried completely for characterization analysis (dark-brown solid), while the second was used for the analysis experiments.

### Photoluminescence measurements

In a typical procedure, the photoluminescence properties of the C-nanoparticles were evaluated by diluting 200 μL of the corresponding C-nanoparticle solution with a pH 6 universal buffer solution (0.2 M Na_2_HPO_4_/0.1 M citric acid) to a final volume of 5 mL. The PL spectra were recorded at 456 nm with excitation at 362 nm for C-dots. For C-onions the PL measurements were taken at 428 nm with excitation at 328 nm. For the detection of metal ions, the reaction mixture was prepared by mixing 200 μL of the corresponding C-nanoparticle solution and 100 μL of metal ion solution (so that the total concentration of metal was 10 ppm) and then adjusting the volume to 5 mL with a universal buffer solution pH 6 (0.2 M Na_2_HPO_4_/0.1 M citric acid). The PL was measured as mentioned above with the same instrumental settings. A 1 cm quartz cuvette was used.

### PL quantum yield measurement

The PL quantum yield was calculated through the well-established comparative method using quinine sulfate as a reference. The following equations were used in the quantum yield measurement:

[2]
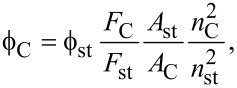


[3]
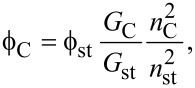


where 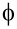 is the quantum yield, *F* is the calculated integrated luminescence intensity, *n* is the refractive index, *A* is the optical density (measured with a UV–vis spectrophotometer, Perkin Elmer, Lambda 900), and *G* is the gradient of a linear plot of *F* as a function of *A*. The subscripts “C” and “st” refer to C-dots (or C-onions) and the reference fluorophore, respectively. Quinine sulphate dissolved in 0.1 M H_2_SO_4_ (*n* = 1.33) with a quantum yield of 0.54 at λ_ex_ = 350 nm was used as a reference. C-dots and C-onions were dissolved in Milli-Q water (*n* = 1.33).

### Instrumentation

HRTEM (JEOL JEM-2100F, 200 kV) was used to determine the size and morphology of the synthesized carbon materials. Powder X-ray diffraction studies were performed on a Bruker D8 Discover instrument with Cu Kα radiation. A Varian 620-IR instrument was used to analyse FTIR spectra on KBr pellets in the range from 600 to 4000 cm^−1^. PL spectra were measured using a Cary Eclipse Varian spectrofluorimeter. UV–vis spectrophotometric analysis was measured with Perkin Elmer, Lambda 900 instrument.

## Supporting Information

File 1 Additional experimental data.

## References

[1] McDonough J K, Gogotsi Y (2013). Electrochem Soc Interface.

[2] Baker S N, Baker G A (2010). Angew Chem, Int Ed.

[3] Esteves da Silva J C G, Gonçalves H M R (2011). TrAC, Trends Anal Chem.

[4] Li H, Kang Z, Liu Y, Lee S-T (2012). J Mater Chem.

[5] Demchenko A P, Dekaliuk M O (2013). Methods Appl Fluoresc.

[6] Hsu P-C, Shih Z-Y, Lee C-H, Chang H-T (2012). Green Chem.

[7] Gaber Ahmed G H, Badía Laíño R, García Calzón J A, Díaz García M E (2015). Microchim Acta.

[8] Gaber Ahmed G H, Badía Laíño R, García Calzón J A, Díaz García M E (2015). Talanta.

[9] Ugarte D (1992). Nature.

[10] Matsumoto N, Mistry K, Kim J-H, Erylmaz O L, Erdemir A, Konoshita H, Ohmae N (2012). Tribol-Mater, Surf Interfaces.

[11] Kuzhir P P, Bychanok D S, Maksimenko S A, Gusinski A V, Ruhavets O V, Kuznetsov V L, Moseenkov S I, Jones C, Shenderova O, Lambin P (2009). Solid State Sci.

[12] Gu W, Peters N, Yushin G (2013). Carbon.

[13] Hantel M M, Presser V, McDonough J K, Feng G, Cummings P T, Gogotsi Y, Kötz R (2012). J Electrochem Soc.

[14] Kuznetsov V L, Chuvilin A L, Butenko Y V, Mal’kov I Y, Titov V M (1994). Chem Phys Lett.

[15] Kuznetsov V L, Chuvilin A L, Moroz E M, Kolomiichuk V N, Shaikhutdinov S K, Butenko Y V, Mal'kov I Y (1994). Carbon.

[16] Cebik J, McDonough J K, Peerally F, Medrano R, Neitzel I, Gogotsi Y, Osswald S (2013). Nanotechnology.

[17] Banhart F (1999). Rep Prog Phys.

[18] Gao Y, Zhou Y S, Park J B, Wang H, He X N, Luo H F, Jiang L, Lu Y F (2011). Nanotechnology.

[19] Yang Y, Liu X, Guo X, Wen H, Xu B (2011). J Nanopart Res.

[20] Zeiger M, Jäckel N, Mochalin V N, Presser V (2016). J Mater Chem A.

[21] Bartelmess J, Giordani S (2014). Beilstein J Nanotechnol.

[22] Walker P L, McKinstry H A, Wright C C (1953). Ind Eng Chem.

[23] Todt M, Bitsche R D, Hartmann M A, Fischer F D, Rammerstorfer F G (2014). Int J Solids Struct.

[24] Suárez-García F, Martínez-Alonso A, Díez Tascón J M (2002). J Anal Appl Pyrolysis.

[25] Smilgies D-M (2009). J Appl Crystallogr.

[26] Short M A, Walker P L (1963). Carbon.

[27] Millikan M, Farrukh M A (2012). Nutritional Metals in Foods by AAS. Atomic Absorption Spectroscopy.

[28] Kokubo K, Matsubayashi K, Tategaki H, Takada H, Oshima T (2008). Nanotechnology.

[29] Chao T-C, Song G X, Hansmeier N, Westerhoff P, Herckes P, Halden R U (2011). Anal Chem.

[30] Shi J, Le Maguer M, Bryan M, Kakuda Y (2003). J Food Process Eng.

[31] Shi J, Le Maguer M, Bryan M, Mazza G, Le Maguer M, Shi J (2002). Lycopene from tomatoes. Functional Foods, Biochemical and Processing Aspects.

[32] Kanasawud P, Crouzet J C (1990). J Agric Food Chem.

[33] Chuvilin A, Kaiser U, Bichoutskaia E, Besley N A, Khlobystov A N (2010). Nat Chem.

[34] Butenko Yu V, Šiller L, Hunt M R C, Sattler K D (2010). Carbon Onions. Handbook of Nanophysics: clusters and fullerenes.

[35] Rao C N R, Subrahmanyam K S, Ramakrishna Matte H S S, Govindaraj A, Pati S K, Enoki T, Rao C N R (2011). Graphene: Synthesis, Functionalization and Properties. Graphene and its fascinating attributes.

