# Supplementary material for: Facile synthesis of water-soluble carbon nano-onions under alkaline conditions
Source: Beilstein J Nanotechnol. 2016 May 27;7:758–66. doi: 10.3762/bjnano.7.67 (PMC4901999; doi:10.3762/bjnano.7.67)
Supplement: File 1 — Additional experimental data. [file Beilstein_J_Nanotechnol-07-758-s001.pdf]

Supporting Information

for

Facile synthesis of water-soluble carbon nano-onions under alkaline conditions

Gaber Hashem Gaber Ahmed[1,2], Rosana Badía Laíño[2], Josefa Angela García Calzón[2] and Marta Elena Díaz García*[2]

Address: [1]Department of Physical and Analytical Chemistry, Faculty of Chemistry, University of Oviedo, c/Julián Clavería, 8. Oviedo, 33006, Spain and [2]Chemistry Department, Faculty of Science, Damanhour University, Damanhour, Egypt

Email: Marta Elena Díaz-García* - medg@uniovi.es

* Corresponding author

Additional experimental data

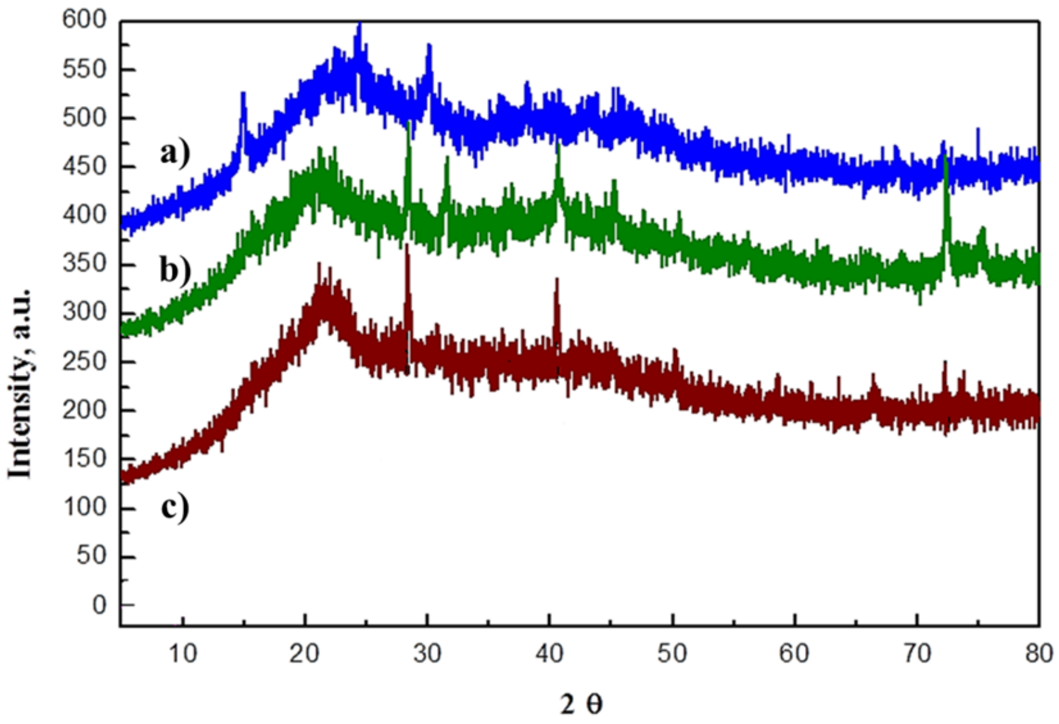

Figure S1: XRD spectra for C-dots obtained by one-pot carbonization of a) carrots, b) tree leaves and c) tomatoes in aqueous media.

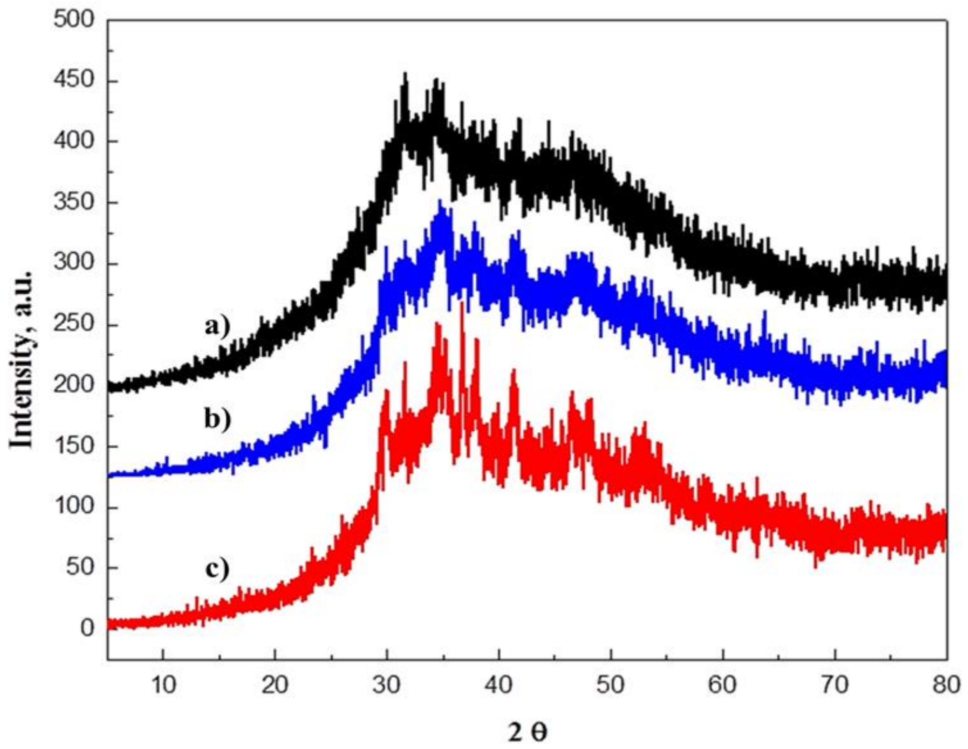

Figure S2: XRD spectra for C-NPs obtained by one-pot carbonization of a) tomatoes, b) tree leaves and c) carrots in NaOH 30% (w/v) media.

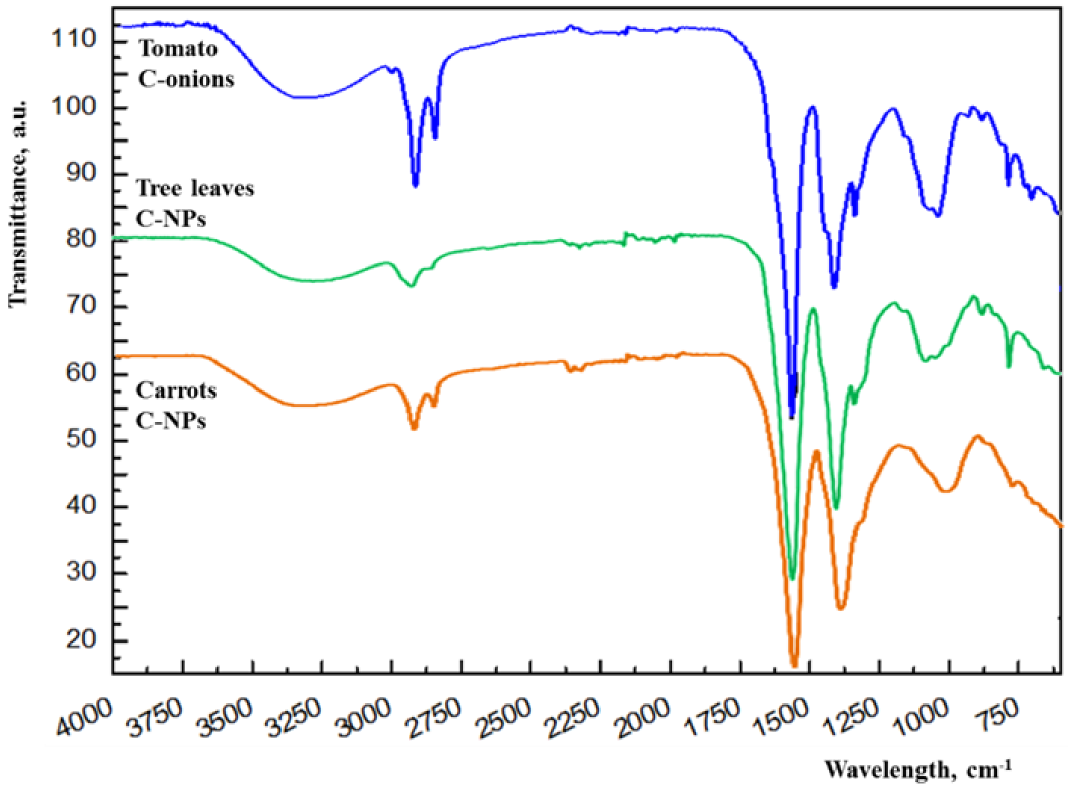

Figure S3: FTIR spectra of C-NPs obtained from tomatoes, tree leaves and carrots by carbonization in NaOH 30% (w/v).

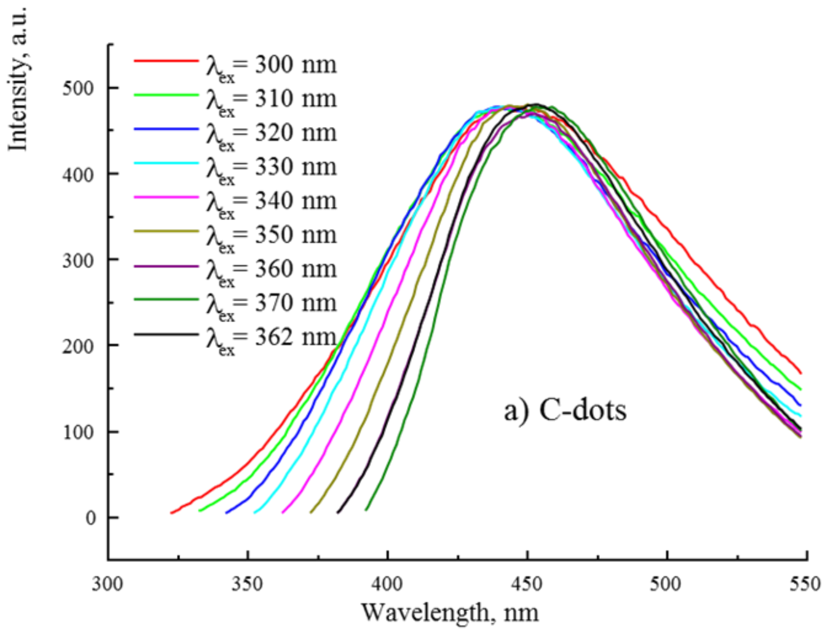

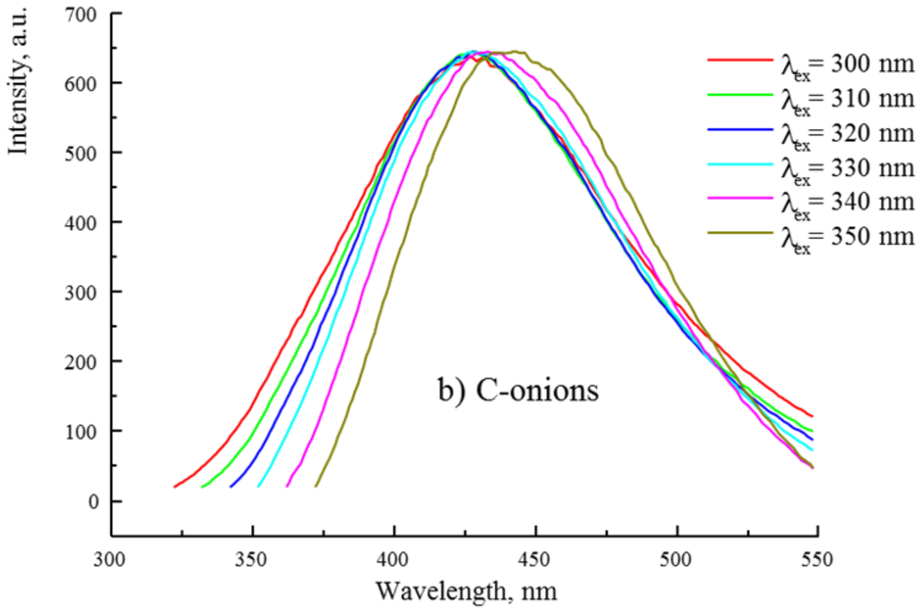

Figure S4: The emission PL spectra of C-NPs obtained from tomatoes under different excitation wavelengths (inset data). a) C-dots obtained in aqueous solution, b) C-onions obtained in 30% (w/v) NaOH. The slit widths of emission and excitation were both 10.

Table S1: Literature values for carbon nanodots and carbon onions obtained from different precursors.

	precursors	quantum yield, ϕ	Ref.
C-dots	citric acid + diethylenetriamine	0.64	[1]
	citric acid + diethylenetriamine + boric acid	0.39	[1]
	citric acid + diethylenetriamine + phosphoric acid	0.70	[1]
	lamp black	0.0087	[2]
	(3-aminopropyl)polyethylene glycol 1500	0.0124	[2]
	tomatoes	0.0132	this work
C-onions	functionalized with BODIPY	0.17	[3]
	functionalized with azomethine ylide	0.08	[4]
	obtained from camphor (burning in low oxygen atmosphere)	0.015	[5]
	obtained from polystyrene foam (burning in low oxygen atmosphere)	0.0165	[5]
	tomatoes + 30% (w/v) NaOH	0.0163	this work

References

[1] Barman M.K.; Jana B.; Bhattcharyya S.; Patra A. **2014**, *J. Phys. Chem. C*, 118, 20034-20041. doi: 10.1021/jp507080c

[2] Bartelmess J; De Luca E.; Signorelli A.; Baldrighi M.; Becce M.; Brescia R.; Nardone V.; Parisini E.; Echegoyen L.; Pompab P.P.; Giordani S. **201**4. *Nanoscale*, 6, 13761–13769. doi: 10.1039/C4NR04533E

[3] Dubey P.; Tripathi K.M.; Sonkar S.K. **2014**, *RSC Adv.,* 4, 5838-5844. doi: 10.1039/C3RA45261A

[4] Georgakilas V.; Guldi D.M.; Signorini R.; Bozio R.; Prato M. **2003**, *J. Am. Chem. Soc.,* 125, 14268-14269. doi: 10.1021/ja0342805

[5] Mao X.J.; Zheng H.Z.; Long Y.J.; Du J.; Hao J.Y.; Wang L.L.; Zhou D.B. **2010**, *Spectroc. Acta Part A- Molec. Biomolec. Spectr.,* 75, 553-557. doi: 10.1016/j.saa.2009.11.015
